# Rodent-Mediated Seed Dispersal Shapes Species Composition and Recruitment Dynamics in Ecotones

**DOI:** 10.3389/fpls.2018.01911

**Published:** 2018-12-20

**Authors:** Fei Yu, Xiaoxiao Shi, Xianfeng Yi, Jianmin Ma

**Affiliations:** ^1^College of Life Sciences, Henan Normal University, Xinxiang, China; ^2^State Key Laboratory of Urban and Regional Ecology, Research Center for Eco-Environmental Sciences, Chinese Academy of Sciences, Beijing, China; ^3^College of Life Sciences, Jiangxi Normal University, Nanchang, China

**Keywords:** ecotone, regeneration, rodent, scatter hoarding, seed dispersal

## Abstract

Ecotones are considered unique environments, and the concepts of edge effects and ecotonal species have been applied widely. Our understanding of the mechanisms that underlie population and community responses to edge effects has been advanced by recent studies. However, little evidence exists to support an increased density and species richness in ecotones regarding rodent-mediated seed dispersal in response to edge plots between communities. *Pinus armandii* and *Quercus variabilis* communities are typical of the Qinling Mountains, China. To elucidate what shapes tree species composition and recruitment dynamics in ecotones, we compared the differences in secondary and tertiary seed dispersal as well as predation in pine and oak by scatter-hoarding rodents as well as the regeneration characteristics of both species in their ecotones with different plots (i.e., 5–8, 15–18, and 27–30 m widths) in the eastern Qinling Mountains. We found that the seeds of pine and oak were removed rapidly, with no differences in the seed removal rates in their ecotones with different plots. Moreover, 13.0 and 36.0% of the scatter hoards of pine and oak, respectively, were established by small rodents in ecotones with a width of 5–8 m, and 3.67 and 7.33% in ecotones with a width of 27–30 m. The seedling densities of pine and oak were significantly higher in ecotones at widths of 5–8 m compared with widths of 15–18 and 27–30 m. According to the seed dispersal and seedling recruitment patterns of pine and oak, the disproportionate abundance of seedlings in ecotones may be due at least partly to patterns of seed caching by rodents.

## Introduction

Seed dispersal is a key process that is responsible for the maintenance and regeneration of plant communities (Vander Wall, 1990; Jansen et al., 2014). Many animals such as rodents and birds determine the diversity and structure of plant communities by caching seeds for future consumption, and then fail to recover or because they died (Vander Wall, 1990; Steele et al., 2014, 2015). Many oak (*Quercus* sp.) and pine (*Pinus* sp.) trees depend on small rodents for seed dispersal in temperate ecosystems (Gómez et al., 2003; Yu et al., 2014) and tropical forests (Xiao et al., 2013). Not all of the buried seeds are retrieved by small rodents and any that escape predation may develop into seedlings in suitable conditions (Gómez et al., 2003; Lu and Zhang, 2004). The burial of seeds may be the most important benefit for plants of rodent seed handling (Briggs et al., 2009). Thus, scatter-hoarding rodents have important roles in the seed-to-seedling phase for plants (Perea et al., 2011; Hirsch et al., 2012; Yu et al., 2017). Various biotic and abiotic factors, such as predation, habitats, and human disturbance, can affect the seed hoarding behavior (caching plant seeds for future consumption) of rodents (Yu et al., 2015, 2017) and seedling recruitment (Smit and Verwijmeren, 2011).

The removal of seeds by rodents directly influences regeneration and colonization in plant populations to affect the plant community structure (Hirsch et al., 2012; Steele et al., 2014; Yu et al., 2014). In particular, cache site selection by scatter-hoarding animals can change due to spatial and temporal variations in the habitat structure as well as competition with conspecifics and other animals, and the perceived risks of predation and pilferage (Muñoz and Bonal, 2011; Hirsch et al., 2012; Steele et al., 2014). Caching seeds in more exposed sites might reduce pilferage and increase the probability of the scatter hoarder being predated, thereby providing an ideal opportunity for seed germination and establishment in more open vegetation (Hirsch et al., 2012; Steele et al., 2014). Rodents are widely distributed in natural forest communities but little is known about the scatter-hoarding behavior of small rodents and their seed dispersal services in response to ecotones. Previous studies have found that the spatial distribution of the scatter hoards made by rodents represents a trade-off between the reduced cache pilferage rates and the increased risk of pilferage (e.g., open canopies) (Steele et al., 2014; Yang et al., 2016). Therefore, determining whether scatter-hoarding rodents select cache sites and then recover their cached seeds in ecotones is an important problem in ecology.

An ecotone is considered to be a transition area between two adjacent habitats and it exhibits different features from those in each habitat because of the interaction between the two environments (Murcia, 1995; Vespa et al., 2014). Ecotones are considered unique environments and many studies have applied the concepts of edge effects and ecotonal species. An edge can be defined as the interaction between two different environments and, in some cases, there can be dramatic biotic and abiotic changes in the habitat area near transitional zones, which may affect some ecological interactions (Mendes et al., 2016). The contact between habitats alters the environmental conditions (edge effects) to possibly influence the population and community attributes, as well as ecological processes (Vespa et al., 2014). One consequence of ecotones have been described as the edge effect, first defined by Odum (1958) as the tendency for increased population density and species richness at the junction zone between two communities. The edge effect described by Odum may occur simply because the ecotone contains representatives of species characteristic of both of the adjacent communities (Baker et al., 2002). Many previous studies have evaluated how the patch size and isolation can affect the population size, abundance, or persistence of species (Prugh et al., 2008; Santos-Filho et al., 2012), whereas few have investigated the effects of landscape changes on key ecological processes such as seed dispersal and seed predation (Mendes et al., 2016).

Ecotones in temperate and tropical forests have a high likelihood of affecting the population dynamics of trees due to abiotic and biotic changes (e.g., by affecting the availability of light and nutrients, and seed predation) (Haurez et al., 2016). The widths of ecotones between communities can greatly affect the vegetation cover and structure of forests, and they are expected to influence animal-mediated seed dispersal and the natural regeneration of plant populations by affecting components of the dispersal process, i.e., detection, colonization, or recruitment (Markl et al., 2012). Edge effects at forest ecotones are recognized as major factors that influence ecological processes (Harper et al., 2005), where they can modify seed dispersal patterns by changing the behavior and abundance of dispersal agents as well as the abiotic conditions (e.g., resistance to predation) (Willson and Crome, 1989). Edge effects have often been investigated by comparing the biodiversity in community and ecotone zones, but few studies have evaluated the differences in animal-mediated seed dispersal and subsequent forest regeneration in two different habitats (Burivalova et al., 2014). Moreover, most previous studies investigated the effects of edges on abiotic factors (Didham and Lawton, 1999), herbivory (Cadenasso et al., 2003), and population and community structures (Ewers and Didham, 2006), whereas few have considered the responses in terms of ecological processes such as seed dispersal (Ingle, 2003; Vespa et al., 2014). However, many previous research focused on how forest fragmentation (which increases edge effects) influences seed dispersal (Morán-López et al., 2015; Aliyu et al., 2018). In addition, previous studies usually focused exclusively on one side of the edge (Restrepo et al., 1999; Cadenasso et al., 2003), or they applied a binary method based on the habitat interior vs. the edge (De Melo et al., 2006). Therefore, the precise mechanisms that allow seed dispersal and establishment to occur in ecotones are poorly understood.

*Pinus armandii* and *Quercus variabilis* communities are the main forest types in the eastern Qinling Mountains, central China. The abundances of both pine and oak seedlings in ecotones are comparable to those in the pine or oak forests (Yu et al., 2013). Similarly, in some tropical and temperate zone forests, the stem densities are higher in ecotones (Matlack, 1994; Yang et al., 2010). Such data have been used to suggest greater emergence and/or survival under ecotones than in other microhabitats. Carolina (1995) noted that this is a type of “direct biological edge effect” where changes in the physical environment caused by edges can directly affect the forest structure. The characteristics of the ecotone have a large impact on both dispersal and plant establishment. Woodland ecotones are extremely heterogeneous, and both seed dispersal and seedling establishment will depend on the physical structure of the ecotone (e.g., the width of the ecotone) (Schupp et al., 1998). The seedlings of both species are rare in mosaic regions when there are large widths between pine and oak forests (personal observations, unpublished data). However, the cause of this phenomenon is unclear and little is known about the contribution of rodent-mediated seed dispersal of these two species to this pattern. In addition, most previous seed dispersal studies only considered primary movement, i.e., the initial movements of seeds from the parent tree, and few investigations have addressed the actual final fate of acorns because of the complex methods required.

Thus, in the present study, we compared the differences in secondary and tertiary seed dispersal as well as predation by scatter-hoarding rodents, and the seedling abundances in ecotones with different plots (i.e., 5–8, 15–18, and 27–30 m widths) between pine and oak communities. We addressed the following question: How do rodent-mediated seed dispersal influence seedling establishment both pine and oak in ecotones? We hypothesized that the abundance of seedlings in ecotones may be due at least partly to patterns of seed caching by rodents. Thus, we aimed to understand ecological processes on rodent-mediated seed dispersal in an ecotone, thereby facilitating improved forest management.

## Materials and Methods

### Study Site

The experiment was conducted on south-facing slopes in the eastern region of Baoan Forest in the Qinling Mountains (109°44^′^–110°40^′^E, 33°52^′^–34°25^′^N), Luonan County, Shaanxi Province, China. The study region is situated in the transitional zone between two macroclimatic regimes (subtropical and warm-temperate zones) with annual precipitation ranging from 950 to 1,200 mm and most occurs between July and September. Snow cover generally lasts for 5 months or more (from November to March). The mean annual temperature is range from 6 to 11°C below 2,000 m and from 1 to 6°C at elevations exceeding 2,000 m above sea level. The forest was harvested during the 1960s and 1970s, and the area is now mainly covered by secondary forests. The secondary forest is dominated by *Q. variabilis* and *P.* *armandii* in the tree layer, and by *Rubus corchorifolius*, *Smilax china*, *Symplocos paniculata*, *Euonymus alatus*, and *Lonicera japonica* in the understory vegetation. The pine–oak mixed forest belt covers about one-quarter of the Qinling Mountains. *Q. variabilis* and *P.* *armandii* forest types were selected because they represented the most common forest types in the region. The forest cover and canopy closure levels both exceeded >90%. An area of approximately 5.0 ha (long-term stand) in the eastern region of the Qinling Mountains was selected as the experimental site. The long-term stand was divided into the three plots with a homogeneous environment, except the width between the communities differed (i.e., distance with 5–8, 15–18, and 27–30 m between the both communities). The three plots were similar in terms of their elevation (1400 m), past land use intensity, tree density (1400–1500 per ha for stems ≥10 cm in diameter at breast height (DBH)). Tree density and main DBH are 1500 per ha and 15–25 cm in oak forest and 1400 per ha and 20–30 cm in pine forest, respectively. No woody plant species with a diameter at breast height (DBH 1.3 m above ground level) >4 cm in ecotones. Only tree seedlings and herbaceous plants distributed in ecotones. The composition of the arboreal component (the two dominant species at all three sites were *Q. variabilis* and *P.* *armandii*). *Apodemus peninsulae*, *Apodemus draco*, and Père David’s rock squirrel (*Sciurotamias davidianus*) are common seed predators in the study region.

### Seed Marking

**FIGURE 1:**
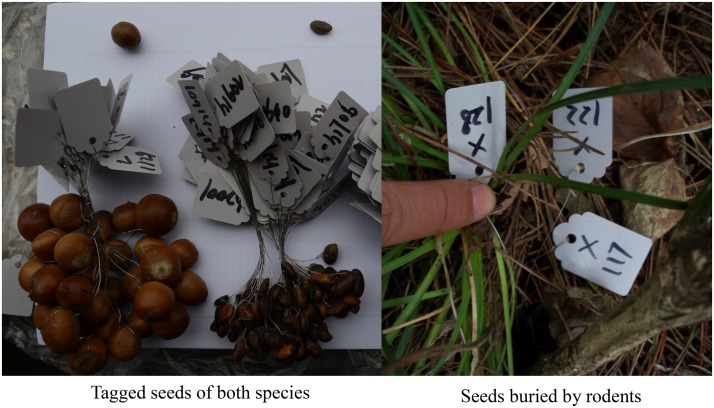
Pictures of tagged seeds of both species and seeds buried by rodents.

Mature, fresh oak and pine seeds were collected from the ground outside the experimental plots for field release. Water flotation and visual inspection were used to distinguish sound and insect-damaged or empty seeds. We randomly selected 900 fresh sound pine seeds (1.30 cm × 0.78 cm, 0.36 ± 0.03 g, *n* = 100) and 900 acorns (1.97 cm × 1.68 cm, 3.58 ± 0.21 g, *n* = 50) (total = 1,800 seeds), and labeled them according to the tin-tagging methods described by Zhang and Wang (2001) and Li and Zhang (2003), with some slight modifications. A hole with a diameter of 0.3 mm was drilled in each seed through the husk near the germinal disk, but without damaging the cotyledon and embryo. A flexible plastic tag (3.0 cm × 1.0 cm, < 0.1 g) was tied through the hole in each seed using thin steel thread with a length of 10 cm. Each seed was marked with a unique numbered tag in order to ensure that the seeds could be easily relocated and identified. Tags frequently remained visible on the surface of the ground after rodents buried the seeds in the soil or litter, and thus they were easy to locate (Figure 1). It has been demonstrated that tagging has negligible effects on the seed removal and hoarding behaviors of rodents (Zhang and Wang, 2001; Xiao et al., 2006).

### Seed Release and Seed Removal

**FIGURE 2:**
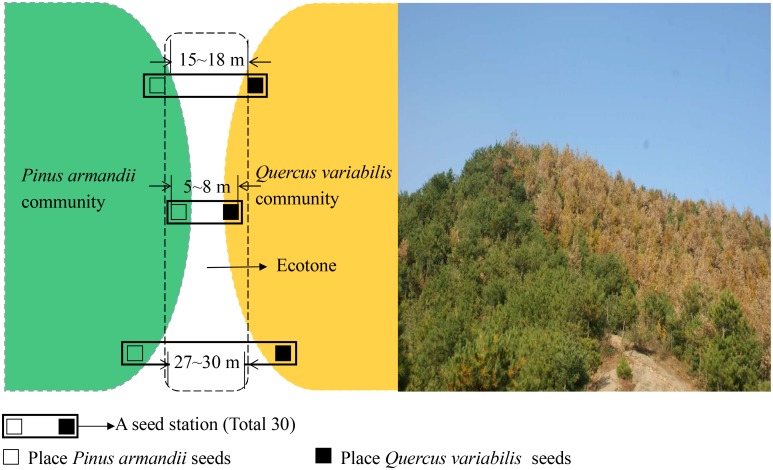
Map showing the locations of the seed stations in the experimental plots.

The large sample site was divided into the three plots based on the width between pine and oak communities (Figure 1), i.e., 5–8, 15–18, and 27–30 m. In each plot, 10 seed stations were established at the forest edges between pine and oak forests, where they were spaced 30 m apart along a transect line (Figure 2). During the seed dropping period in 2015, we placed 30 tagged seeds from each species on the surface at each seed station in separate communities. Each seed station contains both pine and oak seeds. The tagged seeds were placed evenly in the area of the 1 m × 1 m stations.

We checked the fates of the tagged seeds every day at each station immediately after seed release to determine seed harvesting and removal by small rodents. We also randomly searched the area around each seed station at the same time and recorded the fates of the dispersed seeds. The post-dispersal seed fates were classified according to six categories: (1) eaten *in situ* (EIS); (2) moved and eaten where only the plastic tags and seed fragments remained (EAR); (3) intact but not buried after removal (IAR); (4) scatter hoarding in communities (SHC); (5) scatter hoarding in ecotones (SHE); and (6) missing so their true fates were not known. After a cache was discovered, we recorded the seed code numbers and measured the distances between the tagged seeds and their original seed stations, where a chopstick was used to mark the cache’s location and it was coded with the same number as the tag. The sticks were placed 25 cm from each seed cache. During subsequent visits, we also checked all of the caches identified in previous visits until the caches were removed or eaten by rodents. The area around a cache (radius < 50 m) was searched randomly if a marked cache was removed. The fates of the tagged seeds and seed germination (taproot formation) from scatter hoards or seed stations were subjected to surveys at an interval of 1–9 weeks after seed placement in the current year (September 5 to November 4, 2015). The establishment of seedlings from scatter hoards was surveyed during March and May in the following year. The identities of seedlings were determined by checking the numbers on the plastic tags attached to the oak seedling cotyledons.

### Abundance and Species Composition of Small Rodents

In order to identify the rodent species that may have been responsible for removing the released seeds, 48 live steel wire traps (30 cm × 25 cm × 20 cm) baited with peanuts were positioned along three parallel transects at 5-m intervals in each plot during September 10–13, 2015 (immediately before the seed release experiment). The traps were checked two times each day at sunrise and sunset. The captured animals were weighed and then released. The total trapping effort in each plot comprised 48 traps × 3 days = 144. The studies were carried out in accordance with the principles and procedures described in the guidelines for the care and use of laboratory animals as approved by the Henan normal university.

### Selection of Standard Plots and Vegetation Measurements

To determine the regeneration of pine and oak within communities and in ecotones with different plots between communities in the pine–oak forest belt, i.e., 5–8, 15–18, and 27–30 m widths, we set up 20 standard plots measuring 20 m × 20 m with slight human disturbance and similar stand age (approximately 40 years) within the study area. We divided each plot into five subplots measuring 2 m *×* 2 m (100 subplots in total) to measure the seedling densities (excluding sprout regeneration). In total, 40 subplots were established within communities (control plot) and 20 subplots in ecotones. In every subplot, we grouped individuals from each tree species into adults, saplings, and seedlings according to the plant height and DBH (at 1.3 m): adults (DBH > 4 cm), saplings (height > 1 m and DBH < 4 cm when the height exceeded 1.3 m), and seedlings (height < 1 m). All of the seedlings were identified in the subplots to quantify the tree seedling richness for pine and oak. Related parameters including the number of seedlings per plot and coverage were also measured and recorded.

### Data Analysis

Statistical analyses were conducted using SPSS for Windows version 19.0. Cox regression analysis was performed to test for differences in the seed removal rate (the rate at which rodents harvested seeds from the seed stations) among the three plots. A univariate generalized linear model was employed to identify the differences in the seed dispersal distances, the six different seed fates, and seedling regeneration of plots between communities in ecotones. Seeds within each release point within each seed station as a random term in the cox regression and in the generalized linear model analyzing seed dispersal distances and seed fates. The subplots within the plots as a random term in the generalized linear model analyzing seedling regeneration. Tukey’s HSD *post hoc* tests were performed in multiple comparisons of the seed dispersal distances, seed fates, and seedling regeneration between plots. The proportion of rodent trapping success was used to measure the rodent capture rate. A Chi square test was used to compare the differences in rodent capture rate. All of the proportions were arc-sine transformed before their analysis. The proportions compared in the analyses comprised the number of remaining, eaten, and scatter-hoarded seeds, where each was divided by the total number of seeds released.

## Results

### Identification of Seed Removers

We captured 29, 20, and 10 rodents using live traps in the 5–8, 15–18, and 27–30 m plots, respectively (Table 1). In the 5–8 m plot, three small rodent species were trapped over 144 trap nights, i.e., *A. peninsulae* (Muridae) = 72.4% of the captured animals, *Apodemus draco* (Muridae) = 17.2%, and *S. davidianus* (Sciuridae) = 10.3%. In the 15–18 m plot, *A. peninsulae* accounted for 65.0% of the captured animals, *Apodemus draco* for 25.0%, and *S. davidianus* for 10.0%. In the 27–30 m plot, *A. peninsulae* accounted for 80.0% of the captured animals and *A.* *draco* for 20.0%. The capture rate from the 5–8 m plot was significantly higher than that from the 27–30 m plot (*X^2^* = 6.259, *df* = 1, *P* = 0.012).

**Table 1:** Population abundances and rodent species detected in the three experimental plots (*n* = 144 trap days and nights).

Species	Body length (mm)	Body mass (g)	5–8 (m)		15–18 (m)		27–30 (m)	
			Trapped individuals	Trap success (%)	Trapped individuals	Trap success (%)	Trapped individuals	Trap success (%)
*Apodemus peninsulae*	98.2 ± 1.1	24.0 ± 1.2	21	14.6%	13	9.0%	8	5.6%
*Apodemus* *draco*	96.5 ± 1.9	22.7 ± 1.5	5	3.5%	5	3.5%	2	1.4%
*Sciurotamias davidianus*	217.2 ± 5.0	241.5 ± 24.2	3	2.1%	2	1.4%	0	0.0%
Total			29	20.1%	20	13.9%	10	6.9%

### Removal Rates From Seed Stations

**FIGURE 3:**
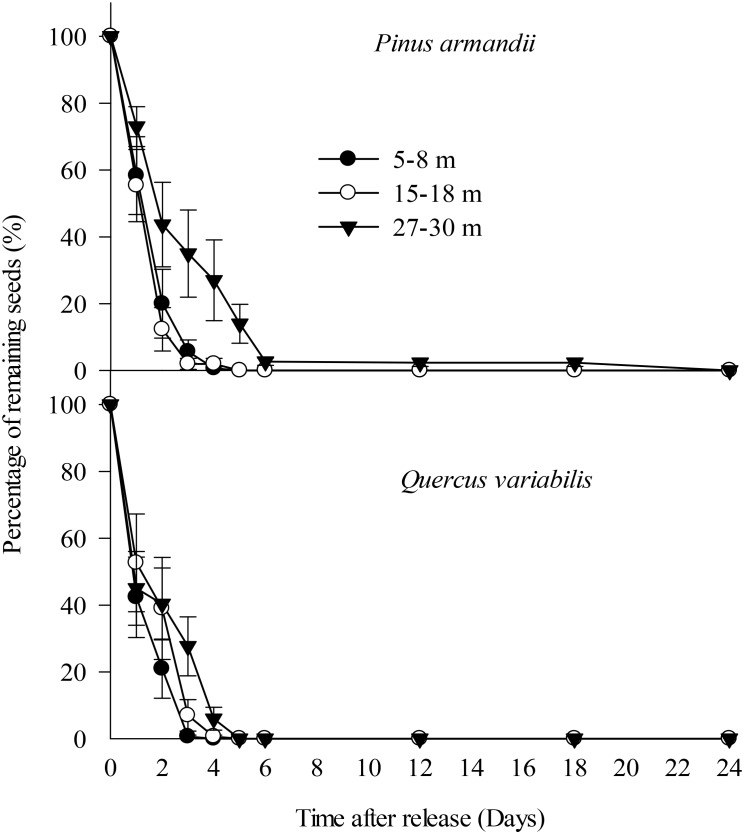
Removal rates for pine seeds and acorns after placement at the seed stations. Data represent the mean ± SE.

All of the *Q. variabilis* acorns released at the seed stations were eaten or removed by small rodents within 5 days of their placement. Similarly, 100.0, 100.0, and 86.0% of the *P.* *armandii* seeds released in the 5–8, 15–18, and 27–30 m plots, respectively, were eaten or removed by small rodents within 5 days of their placement (Figure 3).

The removal speeds for the pine and oak seeds from the 27–30 m plot were slightly lower than those from the 5–8 and 15–18 m plots (Figure 2). However, Cox regression analysis indicated no significant difference in the seed removal rates from the three plots (*Wald* = 2.672, *df* = 2, *P* = 0.263). No significant difference in the seed removal rates between the both species (*Wald* = 3.711, *df* = 1, *P* = 0.054).

### Seed Fates

**FIGURE 4:**
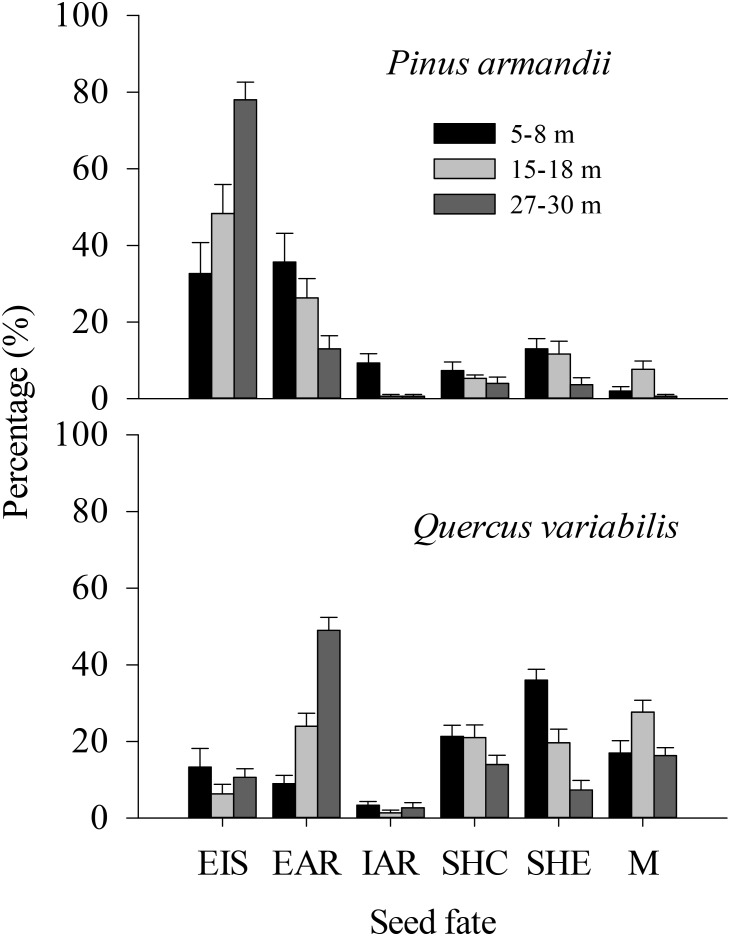
Fates of pine seeds and acorns after primary dispersal by rodents. Data represent the mean ± SE. EIS, eaten *in situ*; EAR, eaten after removal; IAR, intact but not buried after removal to another location; SHC, scatter hoarded within community; SHE, scatter hoarded in ecotone; M, missing; Secondary dispersal: initial movement of seeds from the seed stations.

In total, 13.0% (*n* = 39), 11.7% (*n* = 35), and 3.7% (*n* = 11) of the released pine seeds and 36.0% (*n* = 108), 19.7% (*n* = 59), and 7.3% (*n* = 22) of the released acorns were found in primary caches in the ecotones in the 5–8, 15–18, and 27–30 m plots, respectively (Figure 4). By contrast, 7.3% (*n* = 22), 5.3% (*n* = 16), and 4.0% (*n* = 12) of the released pine seeds and 21.3% (*n* = 64), 21.0% (*n* = 63), and 14.0% (*n* = 42) of the released acorns were found in primary caches within communities in the 5–8, 15–18, and 27–30 m plots, respectively.

The proportions of scatter hoarded in ecotones and intact but not buried after removal seeds were significantly affected by the seed species (SHE: *F* = 8.965, *df* = 1, *P* = 0.004; IAR: *F* = 8.630, *df* = 1, *P* = 0.005) and the plot between communities (SHE: *F* = 8.678, *df* = 2, *P* = 0.001; IAR: *F* = 6.388, *df* = 2, *P* = 0.003) (Figure 4). More seeds were scatter hoarded in ecotones with the 5–8 m width between communities compared with 15–18 m (pine: 13.0 vs. 11.67%; oak: 36.0 vs. 19.67%) (*P* = 0.013) and 27–30 m (pine: 13.0 vs. 3.67%; oak: 36.0 vs. 7.33%) (*P* < 0.001) (Figure 4). Marginally more seeds were scatter hoarded in the ecotone with a width of 15–18 than 27–30 m (*P* = 0.123) (Figure 4).

The eaten *in situ* and scatter hoarded in communities proportions were significantly affected by the seed species (EIS: *F* = 29.078, *df* = 1, *P* < 0.001; SHC: *F* = 34.662, *df* = 1, *P* < 0.001), but not by the plot between communities (EIS: *F* = 0.535, *df* = 2, *P* = 0.589; SHC: *F* = 1.130, *df* = 2, *P* = 0.330) (Figure 4).

More of the pine seeds were eaten *in situ* than the acorns in all plots (32.67% compared with 13.33% at a width of 5–8 m, 48.33% compared with 6.33% at a width of 15–18 m, and 78.00% compared with 10.67% at a width of 27–30 m; *P* < 0.001), whereas more acorns were scatter hoarded (including scatter hoarded in ecotones and scatter hoarded in communities) (SHE: 36.00% compared with 13.00% at a width of 5–8 m, 19.67% compared with 11.67% at a width of 15–18 m, and 7.33% compared with 3.67% at a width of 27–30 m; SHC: 21.33% compared with 7.33% at a width of 5–8 m, 21.00% compared with 5.33% at a width of 15–18 m, and 14.00% compared with 4.00% at a width of 27–30 m; all *P* < 0.001) (Figure 4).

The eaten after removal proportion was not affected significantly by the seed species (EAR: *F* = 1.347, *df* = 1, *P* = 0.251), but there was an effect of the plot between communities (EAR: *F* = 5.609, *df* = 2, *P* = 0.006) (Figure 4).

### Survival of Scatter-Hoarded Seeds

**FIGURE 5:**
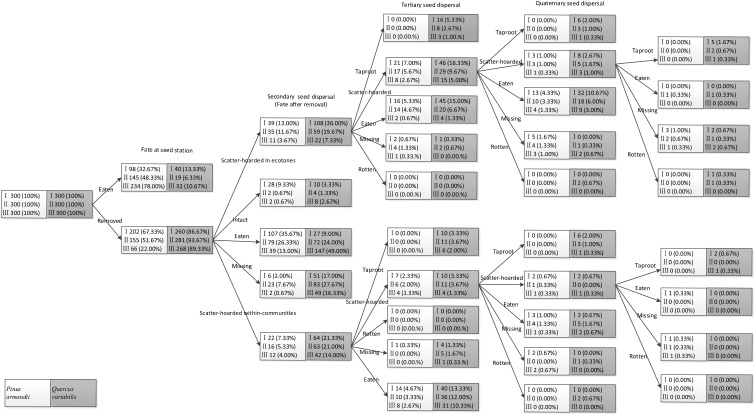
Seed fate pathways for 900 tagged pine seeds and 900 tagged acorns after placement at seed stations. I, II, and III represent ecotone widths of 5–8, 15–18, and 27–30 m, respectively.

Seeds in the primary cache ecotones were less likely to be recovered and they were subjected to lower post-dispersal predation pressure during tertiary and quaternary dispersal than those in the communities (all *P* < 0.001). Consequently, there were significantly more scatter-hoarded seeds in ecotones with a width of 5–8 m than the communities and the plots with the other two widths at 61 days after dispersal (all *P* < 0.001). In total, 6.0% (*n* = 18), 4.7% (*n* = 14), and 2.7% (*n* = 8) of the scatter–hoarded acorns within communities and 9.0% (*n* = 27), 4.3% (*n* = 13), and 1.7% (*n* = 5) in ecotones with widths of 5–8, 15–18, and 27–30 m, respectively, survived until the taproot establishment stage. Among these taproots, only 0.3% (5–8 m: *n* = 1) developed into seedlings within communities and 1.0% (5–8 m: *n* = 3) and 0.3% (15–18 m: *n* = 1) in ecotones. By contrast, taproots and seedlings did not germinate from the tagged pine seeds in all of the plots (Figure 5).

### Seed Dispersal Distance

**FIGURE 6:**
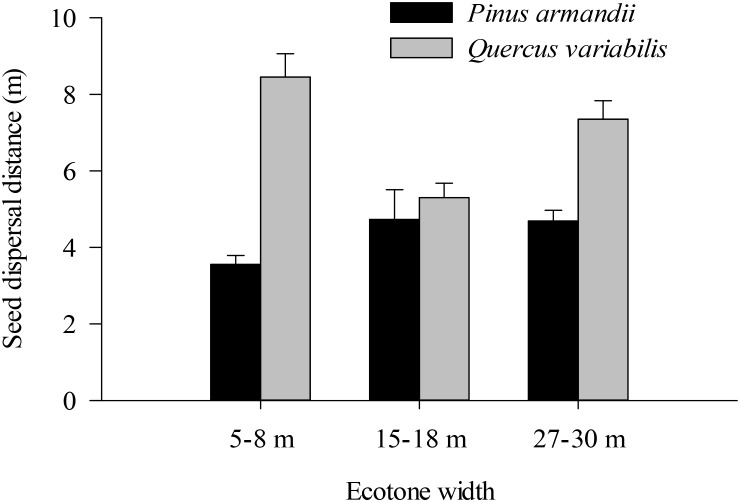
Seed dispersal distances of pine seeds and acorns after their secondary dispersal from seed release stations. Data are expressed as mean ± SE.

Most of the seeds were dispersed at distanced less than 20 m in all of the plots (Figure 6). The average dispersal distance was significantly affected by the seed species (*F* = 61.160, *df* = 1, *P* < 0.001) but not by the plot between communities (*F* = 6.630, *df* = 2, *P =* 0.101). The average dispersal distances of acorns were much greater than those of pine in all plots (all *P* < 0.001).

### Densities of Oak and Pine Seedlings

**FIGURE 7:**
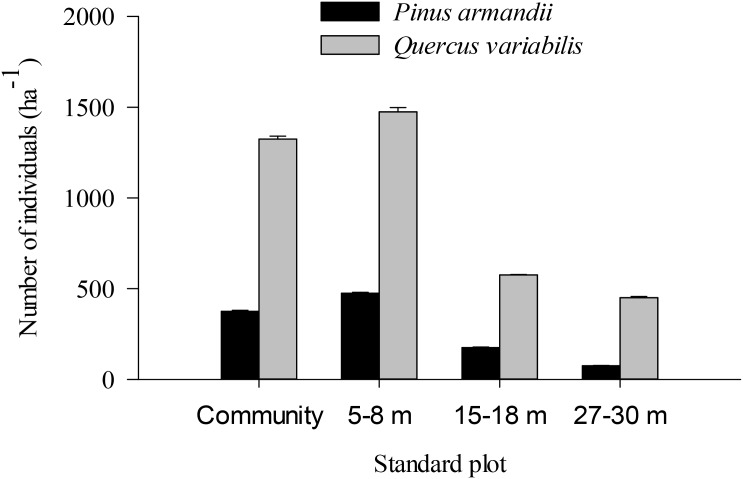
Individual pine and oak seedlings in different plots.

The density of pine seedlings was highest in the 5–8 m plot among the four plots, with 475 stems ha^-1^ (Figure 7). Similarly, the oak seedling density was highest in the 5–8 m plot among the four plots, with 1475 stems ha^-1^. The seedling densities were significantly affected by the seed species (*F* = 20.139, *df* = 1, *P* < 0.001) and plot between communities (*F* = 5.304, *df* = 3, *P =* 0.002) (Figure 7). The seedling density from the pine and oak forests (control plot) was significantly higher than those from the 15–18 m (*P* = 0.028) and 27–30 m plots (*P* = 0.007). Similarly, the seedling density from the 5–8 m plot was significantly higher than those from the 15–18 m (*P* = 0.006) and 27–30 m plots (*P* = 0.001).

## Discussion

### Main Findings

In this study, we found that both pine and oak seeds were removed rapidly, and the seed removal rates did not differ in ecotones with different plots between communities. The pine and oak seeds were rapidly harvested at similar rates from the seed stations, thereby demonstrating the importance of small rodents for dispersing seeds effectively from both tree species. Our results also agree with those obtained in previous studies, which showed that fallen seeds were removed rapidly by rodents (Vander Wall, 1990; Jansen and Forget, 2001; Yu et al., 2017). Moreover, 13.0 and 36.0% of the scatter hoards of pine and oak, respectively, were established by small rodents in ecotones with a width of 5–8 m, and 3.67 and 7.33% in ecotones with a width of 27–30 m. The seedling densities of pine and oak were significantly higher in ecotones at widths of 5–8 m compared with widths of 15–18 and 27–30 m. Our findings support the hypothesis that the abundance of seedlings in ecotones may be due at least partly to patterns of seed caching by rodents.

### Seed Fates and Seed Dispersal Distances

The rodents exhibited no overall caching preference between the communities and ecotone habitats in our study. We found different seed dispersal patterns in the ecotone plot with a width of 5–8 m compared with the other two types of ecotone plots and within communities. In contrast to the ecotone plot with a width of 5–8 m, we recorded lower proportions of scatter-hoard seeds within communities and ecotone plots with widths of 15–18 and 27–30 m. Our results clearly showed that a larger width between communities reduced seed cache placement in the ecotones. Previous studies have assumed that the spatial distribution of scatter hoards leads to a simple tradeoff between the benefits of spacing caches and the cost of retrieving caches with wider spacings (Steele et al., 2015; Yang et al., 2016). There are two key processes: (a) rodents might be under greater predation risk in wider, more exposed areas; (b) spacing caches over a greater spatial extent might be costly for retrieval. Both (a) and (b) could be occurring in this system and might interact. Therefore, the ecotone appeared to be a key determinant of the microhabitat heterogeneity in the forest understory and it affected the activity and foraging behaviors of rodents, as well as seed germination and seedling establishment. The ecotone plot with a width of 5–8 m favored fast-growing herbaceous plants, e.g., grasses, especially in areas with an open canopy (personal observation). Previous studies have shown that edge effects depend on features of the surrounding landscape, such as the percentage of forest cover (Robinson et al., 1995; Hartley and Hunter, 1998) and the degree of contrast in the vegetation structure between the patch and surrounding matrix (Ries et al., 2004). It is possible that the increased vegetation in the ecotone plot with a width of 5–8 m contributed to greater abundances of small rodents, thereby increasing the amount of scatter hoards as well as seed dispersal (Lambert et al., 2005).

Small rodents exhibited no preference for ecotones when establishing their caches, but seeds in the primary caches in ecotones were recovered less frequently and they were under lower post-dispersal predation pressure during the tertiary and quaternary dispersal processes compared with those within the communities. We found that the retrieval of scatter-hoarded seeds from ecotones by small rodents was rare. Edge effects might modify the patterns of seed dispersal by changing the behavior of dispersers and the environmental conditions (Vespa et al., 2014). The higher activity (including seed predation) of small rodents under the vegetation cover may be explained by the increased risk of predation for rodents in relatively open habitats (Lu and Zhang, 2004). Our results suggest that the risk of predation for rodents may affect their seed-hoarding behavior (Li and Zhang, 2003; Lu and Zhang, 2004) and the subsequent recruitment of seedlings (Duncan and Chapman, 1999).

Previous studies have indicated that small seeds have a higher likelihood of being eaten *in situ*, whereas larger seeds such as acorns are more likely to be cached for future use by rodents (Vander Wall, 1990; Jansen and Forget, 2001). Rodents may find large seeds more attractive as food reserves for caching (Jansen and Forget, 2001; Jansen et al., 2002). Our results clearly demonstrated that pine seeds had a greater likelihood of being eaten *in situ*, whereas more acorns were scatter-hoarded by rodents. We found that the roles of rodents in seed removal varied among plant species but they were mainly dependent on seed size (Price and Jenkins, 1986). More information is required regarding the specific effects of seed traits on seed dispersal in ecotones, although it has been shown that large acorns have a greater likelihood of being cached in open habitats by scatter-hoarding animals (Steele et al., 2014).

Our results showed that the seed dispersal distances were significantly influenced by the seed species rather than the width between communities. The average dispersal distances were much greater for acorns than pine seeds in all plots, possibly due to the difference in seed size. The results obtained in the present study support the hypothesis of Jansen et al. (2002) who proposed that larger seeds will dispersed a greater distance from their parent trees (or seed stations). However, the effect of the width between communities on the dispersal distance was not as high as expected. The distance of release points into the ecotone might also affect the distance of dispersal and seed fate.

### Seedling Regeneration

Only a small number of oak seedlings germinated from the tagged seeds in our study area and no seedlings germinated from the tagged pine seeds in all plots. Our results agree with those obtained in previous studies where only 0.02–10% of the removed seeds had the capacity to establish seedlings (Chang et al., 2012; Yu et al., 2014, 2015). However, both species are known to be successfully regenerated and recruited in the field in the Qinling Mountains (Yu et al., 2013). Three possible explanations might explain this inconsistency: (1) some seeds may have been scatter hoarded at locations outside the study area to establish seedlings; (2) high populations of rodents together with a low seed crop during 2015 as well as severe drought during the spring in 2016; and (3) negative effects on seed germination and seedling establishment of seed tagging. Many studies have shown that mast seeding affects the seed dispersal strategies of rodents (Jansen and Forget, 2001; Chang et al., 2012). For example, Li and Zhang (2007) have reported that mast seeding increased proportion of scatter hoarding and dispersal distance. Also, the cached seeds may damage by winter desiccation or most seedling deaths were caused by summer desiccation (Vander Wall, 1994; Chang et al., 2012). Therefore, although the seedling proportions were not high, the rodent caching behavior probably contributes significantly to seed survival and the establishment of seedlings because tens of thousands of seeds are produced by each tree during every year under natural conditions (Chang et al., 2012). If we assume that the low cache dynamics by small rodents in the plot with a width of 27–30 m between communities is a general pattern, then this could compensate for the high mortality due to seed predation by small rodents.

### Key Seed Removers

The numbers of seeds that are dispersed or scatter hoarded depend on the composition and abundance of the dispersers and the number of seeds removed by each disperser (Jansen and Zuidema, 2001). Our observations agree partly with those obtained in previous studies where the composition and richness of mammal species was significantly affected by the habitat structure and forest edges (Burivalova et al., 2014; Steele et al., 2014). We did not capture *S. davidianus* in the plot with a width of 27–30 m between the communities, mainly because this large-bodied disperser is frequently more susceptible to disturbance (Terborgh et al., 2008; Holbrook and Loiselle, 2009). We cannot fully exclude the potential roles in seed removal of different animals (e.g., wild pigs, cattle, Eurasian jays, and pheasants) in addition to small rodents, although their effects might be negligible compared with small rodents because of their very low abundances in the study area (Chang et al., 2012; Yu et al., 2013). In particular, we have found that jays rarely pick up seeds from the ground, but rather directly from trees in the middle Qinling Mountains (Personal observation). However, no jay was found in the eastern Qinling Mountains during the experiment period. Thus, the actual dispersers were mainly small mammal dispersers.

In summary, our results indicate that disproportionate abundance of seedlings in ecotones may be due at least partly to patterns of seed caching by rodents. We also found that ecotonal width was important for determining whether seeds were removed and cached by predators or potential dispersers that affected seed fate and tree regeneration. Ecotones probably substantially modify the compositions of granivorous communities as well as affecting seed dispersal services and the capability of plant movement, which might contribute to edge effects. Thus, the plant–animal interactions modified by ecotones should be considered in small-scale forest management projects or research. Our results are of directly importance for the Qinling Mountains but they might have broader implications for pine–oak forests in other parts of the world and other systems. There was only one true replicate per type of ecotone width that might hamper the generalization of the results due to the limitation of experimental condition. The larger plots occur at the edges of the forest, so the surrounding matrix habitat of those forests (i.e., grassland) might conflate ecotone width effects with other environmental factors. Further research is required to understand the effects of ecotones and different ecological factors on seed-caching animals (including birds), and the possible influence of the seed rain intensity into ecotones with different widths on the reproductive success of plants over diverse scales in space and time.

## Author Contributions

FY, XY, and JM conceived and designed the experiments. FY and XS analyzed the data and wrote the paper. All authors performed the experiments and read and approved the final manuscript.

## Conflict of Interest Statement

The authors declare that the research was conducted in the absence of any commercial or financial relationships that could be construed as a potential conflict of interest.
